# Supplementary material for: Visualizing omicron: COVID-19 deaths vs. cases over time
Source: PLoS One. 2022 Apr 19;17(4):e0265233. doi: 10.1371/journal.pone.0265233 (PMC9017933; doi:10.1371/journal.pone.0265233)
Supplement: Attachment — Submitted filename: Response_to_Reviewers.docx [file pone.0265233.s001.docx]

We thank the Editor and the Reviewers for their constructive comments. Please find point-by-point responses below.

**Journal Requirements:**

1. Please ensure that your manuscript meets PLOS ONE's style requirements, including those for file naming.

*We have made the style updates.*

2. PLOS requires an ORCID ID for the corresponding author in Editorial Manager on papers submitted after December 6th, 2016. Please ensure that you have an ORCID iD and that it is validated in Editorial Manager.

*Both authors have registered our ORCID iDs in PLOS.*

3. Please review your reference list to ensure that it is complete and correct…Any changes to the reference list should be mentioned in the rebuttal letter that accompanies your revised manuscript.

*We have reviewed the reference list. It appears there is a newer citation replacing:*

Chan MCW, Hui KP, Ho J, Cheung M, Ng K, Ching R, et al. SARS-CoV-2 Omicron variant replication in human respiratory tract ex vivo 2022. <https://doi.org/10.21203/rs.3.rs-1189219/v1>.

*with:*

*Hui KPY, Ho JCW, Cheung M-C, Ng K-C, Ching RHH, Lai K-L, et al. SARS-CoV-2 Omicron variant replication in human bronchus and lung ex vivo. Nature. 2022. doi:10.1038/s41586-022-04479-6*

*We have removed (not required):*

*Cameroni E, Saliba C, Bowen JE, Rosen LE, Culap K, Pinto D, et al. Broadly neutralizing antibodies overcome SARS-CoV-2 Omicron antigenic shift. 2021.*

**Reviewers' comments:**

Reviewer #1:

“This publications accesses publicly available information on COVID cases and deaths and utilizes a novel type of graphic display to illustrate the dynamics of the infection. It includes a large number of fascinating graphs which show differences in the different waves of COVID.”

*Thank you. We agree that this is an interesting and useful way to visualize the dynamics of severity and transmissibility of infection.*

“In using the plots to compare countries, as stated in the discussion, it's important to realize that both X and Y axes are scaled differently from graph to graph; I think it would be valuable to say this prior to the detailed country-by-country discussion for the readers' benefit.”

*We have now noted this prior to the country-by-country results.*

“I wonder if it's possible to make the discussion more specific about how to utilize this type of graph to illuminate causative epidemiological variables…Would it make sense to pick a country or two, display cases vs deaths graphs side-by-side with more-conventional representations, and discuss the differences in information conveyed? Right now, the paper seems slightly epiphenomenal; these are cool graphs, but it's a bit unclear exactly what makes them distinctively useful.”

*We appreciate this point and have now included Fig 9, which picks three countries (Denmark, Finland, France) and displays the time series side by side with the bivariate plots, to illustrate the dynamics more clearly/easily visible in bivariate plotting.*

Reviewer #2:

In this paper, the authors have utilized an uncommon COVID-19 graph plotting system for visualizing the pandemic data…I see no major issues and one minor issue. On page 4, results section, 1st paragraph, and 2nd sentence, It looks as though the author wanted to state “total death vs. total cases” and not “total death vs. total death.” Overall, I accept the publication with one minor corrections since the paper meets all seven criteria set by the journal.

*Thank you for this summary and for pointing out the typo. We have now fixed it.*
